# Sustainable Healthcare: A Perspective on the Current Landscape, Frameworks, Barriers, and Implementation Model in a Quaternary Hospital System

**DOI:** 10.3390/ijerph23081028

**Published:** 2026-08-06

**Authors:** Hubert Tuyishime, Carole Lin, Amandeep Chawla, Bryan Khoo, Kevin G. Shea

**Affiliations:** 1 Department of Orthopaedic Surgery, Stanford University School of Medicine, Stanford, CA 94305, USA; thubert@stanford.edu (H.T.); bryankhoo37@gmail.com (B.K.); 2 Department of Anesthesiology, Perioperative and Pain Medicine, Stanford University School of Medicine, Stanford, CA 94305, USA; clin999@stanford.edu; 3 Department of Supply Chain and Post Acute Care, Stanford Healthcare, Stanford, CA 94305, USA; achawla@stanfordhealthcare.org

**Keywords:** emission reduction, healthcare sustainability, supply chain, decarbonization, waste reduction

## Abstract

**Highlights:**

**Public health relevance—How does this work relate to a public health issue?**

- Healthcare services account for 4–5% of global greenhouse gas emissions and generate plastic and chemical waste that contribute to environmental pollution and climate-related health hazards.
- Health-related hazards include microplastic ingestion, respiratory and cardiovascular diseases, and malnutrition, which disproportionately affect low-resource, vulnerable, and marginalized communities.

**Public health significance—Why is this work of significance to public health?**

- Despite growing decarbonization frameworks, integrating sustainability measures into healthcare and supply chain systems remains challenging due to limited leadership buy-in, misaligned financial incentives, and inadequate regulatory accountability.
- Stanford Medicine’s initiatives illustrate how a large health system may pursue emissions and waste reduction while maintaining high-quality care and lowering costs, and are offered as an implementation example.

**Public health implications—What are the key implications or messages for practitioners, policy makers and/or researchers in public health?**

- Healthcare institutions should integrate sustainability into operations, supply chain design, and clinical workflows to minimize their contribution to environmental pollution.
- Collaboration with governments, advocacy and research organizations, industry partners, and impacted communities is essential to standardize sustainability requirements, scale implementation, and ensure accountability across stakeholders.

**Abstract:**

Healthcare services account for approximately 4–5% of global greenhouse gas emissions worldwide, and nearly 10% of emissions in the United States are primarily from general hospital operations, surgical and anesthetic care, and pharmaceutical and medical device supply chains. Healthcare services and operations also generate substantial plastic and chemical waste that pollute the environment and disproportionately affect vulnerable communities. Although global and national organizations have introduced decarbonization frameworks, adoption remains hindered by limited leadership engagement, competing institutional priorities, and insufficient regulatory accountability. This article synthesizes the published literature on the current landscape of healthcare sustainability, summarizes key policy frameworks, and identifies major barriers to implementation; these findings are then illustrated through sustainability initiatives at Stanford Medicine, a quaternary hospital system. These efforts are presented as implementation examples that other institutions may adapt to their own resources and contexts, to reduce emissions and ensure more sustainable healthcare delivery.

## 1. Introduction

The foundational medical principle of ‘first, do no harm,’ rooted in the Hippocratic tradition of protecting patient welfare, has evolved to encompass environmental stewardship, reflecting growing recognition that clinicians bear responsibility not only for individual patients but for the communities and ecosystems in which those patients live [1,2,3]. Yet, healthcare services account for approximately 4–5% of total greenhouse gas emissions worldwide, and up to 10% in the United States [4,5]. The largest contributors of greenhouse gas emissions include hospitals, surgical services, and, most importantly, pharmaceutical and medical supply chains. Some hospital emissions stem from intensive energy requirements for heating, cooling, and powering medical equipment [6]. Surgical and procedural services, often characterized as “carbon hotspots” in hospital-based care, with a plethora of materials and packaging of medical supplies used in clinical care, necessitate the use of anesthetic gases and maintenance of sterile environments, which may contribute to emissions [7,8]. Pharmaceutical and medical supply chains, estimated to contribute over 50% of total healthcare emissions, are particularly large contributors through the process of taking products from manufacturing, to transportation, then ultimately to disposal [9]. Beyond carbon emissions, health care systems also produce large volumes of plastic and chemical waste that contribute to environmental pollution, loss of biodiversity, and climate-related health hazards [10]. Children, minority groups, and other vulnerable populations bear the greatest burden of these environmental effects [11]. Concerted efforts are needed from healthcare institutions to lead initiatives toward decarbonization and sustainability.

Previous sustainability efforts in healthcare often focused on narrow domains such as facility design, recycling, or isolated energy-efficiency projects. Although valuable, these strategies are siloed and consequently have limited effect, particularly when the majority of healthcare’s carbon footprint originates from complex, interdependent supply chains [12]. Recent policy and research initiatives such as the Global Roadmap for Healthcare Decarbonization [13], the World Health Organization (WHO) Sustainable Health Systems Guidance [14], the United States Agency for Healthcare Research and Quality (AHRQ) Primer on Reducing Healthcare Carbon Emissions [15], and the Society of American Gastrointestinal and Endoscopic Surgeons (SAGES) and European Association for Endoscopic Surgery (EAES) joint taskforce review of sustainability in surgical practice [16] highlight and provide implementation frameworks to address the need for integrated sustainability practices across healthcare systems.

Despite the rise in sustainability frameworks and the research literature, the implementation of sustainable healthcare delivery remains challenging. A notable barrier includes the paucity of sustainability champions across organizations to drive policy changes in alignment with global and national sustainability guidelines [17,18]. Furthermore, misaligned financial priorities, lack of institutional focus, limited training for clinicians and staff, and the lenient enforcement of sustainability performance by government or regulatory organizations hinder possibilities for change toward more sustainable healthcare delivery [19]. Academic medical centers are uniquely positioned to address these limitations through innovation, research, and multidisciplinary collaborations, although smaller, private systems may have room to innovate in this area as well.

While the peer-reviewed literature addresses specific sustainability solutions, there remains a need for an integrated, multidisciplinary implementation model at the institutional level. This article addresses that gap by combining the peer-reviewed literature, policy and framework documents, and a descriptive review of the published literature and institutional reporting from sustainability efforts at Stanford Medicine, demonstrating practices across governance, supply chain, and clinical domains within a large U.S. quaternary—providing highly specialized and advanced care—health system. This article is guided by two research questions: (1) What sustainability strategies have demonstrated effectiveness in the published literature, and what barriers continue to impede broader adoption? (2) How have integrated governance, supply chain, and frontline engagement strategies been operationalized across multiple domains within a large, complex academic health system, and what outcomes have been documented?

## 2. Previous Literature on Sustainability: Current Landscape, Frameworks and Barriers

### 2.1. Current Landscape

**Figure 1:**
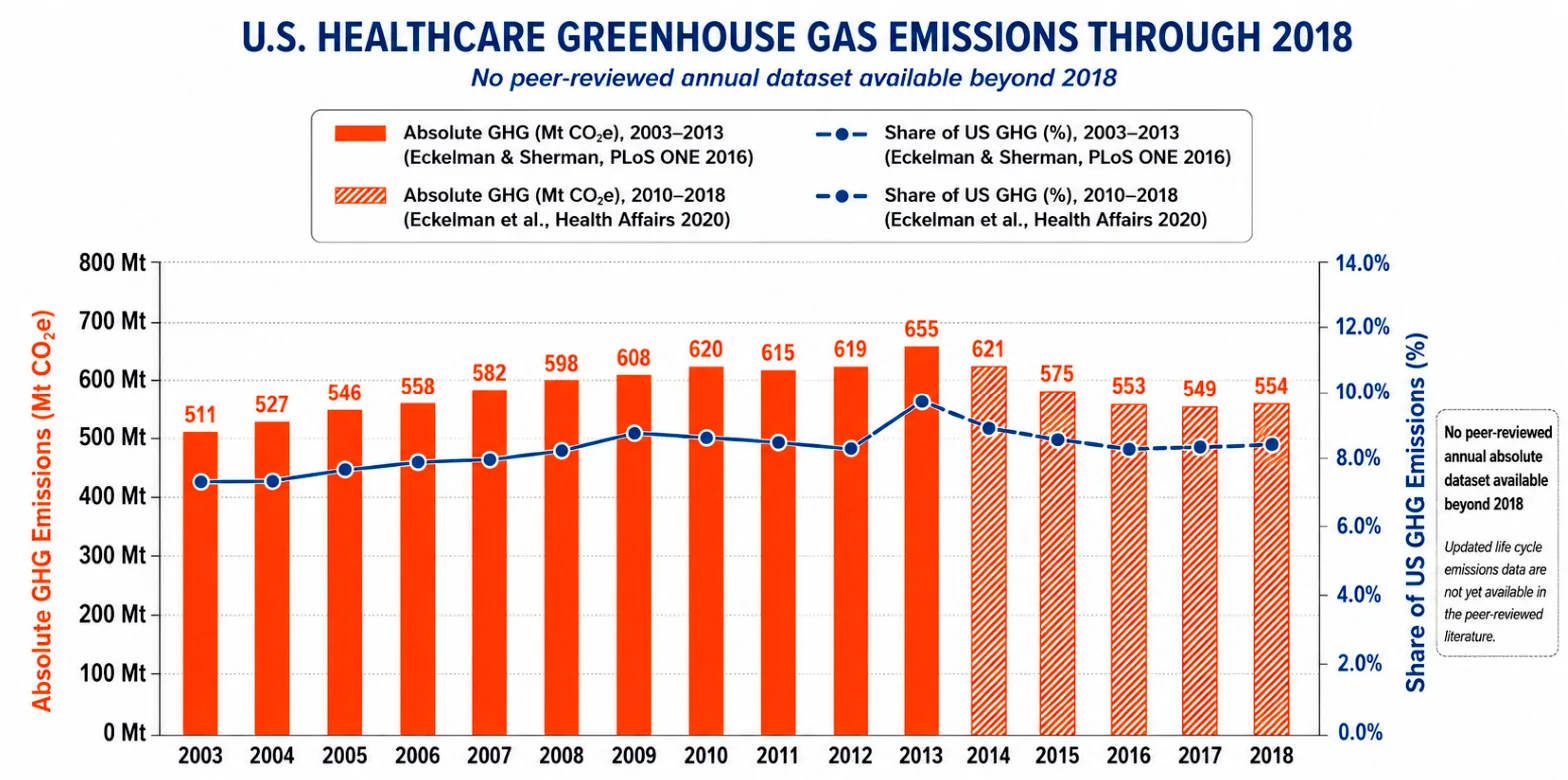
Time series of life cycle greenhouse gas emissions from US health care activities. Figure 1 demonstrates that U.S. healthcare greenhouse gas emissions increased substantially from 2003 to 2013 and remained persistently high through 2018, highlighting the need for sustained decarbonization efforts. Data for 2003–2013 derived from Eckelman and Sherman 2016 [21]; data for 2010–2018 derived from Eckelman et al. [27], demonstrating a rise in U.S. healthcare greenhouse gas emissions to 554 Mt CO_2_e by 2018. No peer-reviewed annual absolute emissions dataset for U.S. healthcare is currently available beyond 2018. Mt CO_2_e = million metric tons carbon dioxide equivalent. The figure image resolution and color contrast were enhanced utilizing ChatGPT images 2.0.

Healthcare is one of the most carbon- and resource-intensive industries worldwide, with a significant burden on the environment and population health. The healthcare industry produces approximately 2250 Mt CO_2_e per year, which equates to the previously reported 4–5% of total global greenhouse gas emissions, and nearly 10% of greenhouse gas emissions in the United States [20,21] (Figure 1). If considered a nation, the U.S. healthcare system would rank as the fifth-largest emitter in the world [20]. Hospitals account for nearly one-third of total healthcare emissions, largely due to the continuous operation of heating, ventilation, and air-conditioning systems required to maintain sterile environments [6]. Surgical and procedural rooms are particularly carbon-intensive, with operating rooms (ORs) using three to six times more energy per square foot than other hospital spaces and generating 20–30% of total hospital waste [18,22,23,24]. Additionally, anesthetic gases with high global warming potential, such as desflurane and nitrous oxide, contribute up to 74% of the operating room’s carbon output, with warming potentials up to 3700 times greater than carbon dioxide [25,26].

**Figure 2:**
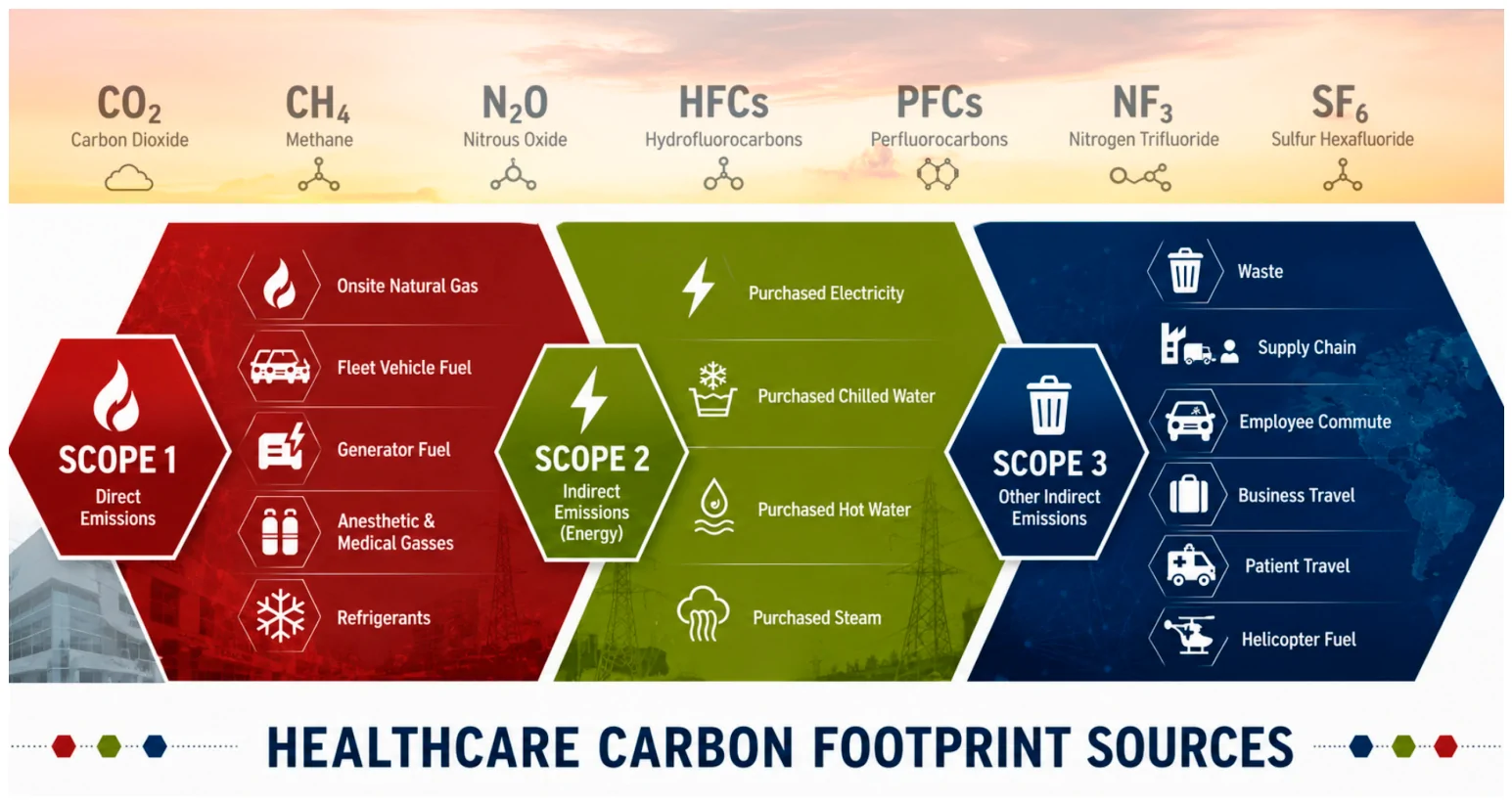
Types of emissions are categorized into three scopes. Scope 1 emissions are direct emissions from on-site sources (e.g., boilers, fleet vehicles, refrigerant or anesthetic gas leakage). Scope 2 covers emissions from purchased energy, such as electricity or externally supplied chilled or hot water. Scope 3 includes all other indirect upstream and downstream emissions (e.g., waste or supply chain). The largest component of healthcare emissions lies in Scope 3 activities. The figure image resolution and color contrast were enhanced utilizing ChatGPT images 2.0.

Beyond hospitals, healthcare’s Scope 3 supply chain emissions (Figure 2), those generated by purchased goods, pharmaceuticals, and medical devices, represent over 70% of the health sector’s carbon footprint [9,28]. Pharmaceuticals and device production, transportation, and disposal account for 50–60% of total healthcare-related emissions [6]. Single-use instruments and sterile packaging materials can produce up to 10 kg of daily waste per hospital bed [18,29] and up to 12 kg of waste per operating-room case [30,31], contributing to the growing waste burden. For example, the US generates 5 million tons of waste annually, with less than 10% being recyclable [24]. Persistent plastics degrade into micro- and nanoplastics, which have been found in critical human tissue and organs, including in the blood [32,33], lungs [34], and placental tissue [35,36]. This raises concerns about potential effects on human health, including inflammatory, endocrine, cardiovascular, and developmental toxicities [37,38,39].

Beyond consequences on an individual patient’s health, the environmental consequences of unsustainable healthcare practices extend directly to population health. The WHO identifies climate change as the greatest health threat of the 21st century, projecting an additional 250,000 deaths annually between 2030 and 2050 from malnutrition, malaria, diarrhea, and heat-related illness [40,41]. Rising global temperatures, extreme weather events, and air pollution are already disrupting hospital operations and supply chains while worsening respiratory and cardiovascular diseases [42,43,44]. These effects disproportionately impact vulnerable populations, exacerbating rates of malnutrition, malaria, and diarrhea in children in low-income countries, increasing heat exposure in elderly people and flooding marginalized communities in remote areas [40,41,45]. Concerted efforts are needed to design and implement sustainable models of healthcare delivery and limit the exacerbation of climate change consequences on human and planetary health.

### 2.2. Frameworks and Initiatives

#### 2.2.1. Global Frameworks

Multilateral organizations and global advocacy agencies have called for urgent efforts to improve sustainability across health systems. The WHO’s report, Safe, Climate-Resilient and Environmentally Sustainable Health Care Facilities: An Overview (2024), provides guidance on building safe, climate-resilient and environmentally sustainable health care facilities and actionable strategies to implement them [14]. This report aligns with the Health Care Without Harm’s report, Global Roadmap for Health Care Decarbonization: A Path to Zero Emissions (2021), which quantifies healthcare’s emission contribution at 4.4% of global net emissions, ranking the sector among the top five emitters worldwide [46]. The Roadmap sets a goal of net-zero emissions by 2050 and defines three strategic decarbonization pathways: (1) healthcare operations, (2) supply chain, and (3) wider economy and society. It also provides seven high-impact actions, including: (1) 100% renewable electricity, (2) zero-emission buildings and infrastructure, (3) zero-emission travel and transport, (4) healthy and sustainably grown food, (5) low-carbon pharmaceuticals, (6) circular health care waste management, (7) healthy system effectiveness. Implementation of this Roadmap at scale alone could prevent 44.8 gigatons of CO_2_-equivalent emissions between 2014 and 2050 [46].

International specialty and clinical societies have translated these high-level frameworks into discipline-specific guidance to drive measurable change. The Society of American Gastrointestinal and Endoscopic Surgeons (SAGES) and the European Association for Endoscopic Surgery (EAES), in their Scoping Review for the SAGES and EAES Sustainability in Surgery Task Force (2024), proposed the 10R Circular Framework: Refuse, Reduce, Rethink, Reuse, Repair, Refurbish, Remanufacture, Repurpose, Recycle, and Recover to standardize sustainability across surgical practices and perioperative care [16]. This model aims to minimize waste, optimize resource utilization, and ensure sustainable production of tools and equipment. Similarly, the World Federation of Societies of Anesthesiologists (WFSA)’s 2021 consensus on Principles of Environmentally Sustainable Anesthesia recommends: eliminating high global warming potential anesthetic agents (desflurane and nitrous oxide), reducing waste, using low-flow anesthesia, and instituting anesthetic gas recovery systems [47].

#### 2.2.2. United States-Focused Initiatives

In the United States, the Agency for Healthcare Research and Quality (AHRQ) has led the national policy response through its primer on Reducing Healthcare Carbon Emissions (2022) [15]. This primer offers prioritized measures and concrete examples that organizations may use to target their decarbonization goals with aims spanning energy, transportation, anesthetic gas, pharmaceuticals and chemicals, medical devices and supplies, and food. It provides flexibility, with measures that can be tailored to each institution’s present state and future goals. Additionally, the US Environmental Protection Agency (EPA) has set forth a blueprint for managing pharmaceutical waste in US healthcare facilities [48]. The Joint Commission has also established a sustainable healthcare resource center to provide guidance to hospital programs and instituted a certification program to qualify organizations by assessing their leadership and commitment to sustainability, measurement of data to track progress, and performance improvement in transportation, buildings and energy use, anesthetic gases, and waste [49]. Clinical and professional societies, such as the American College of Surgeons, published a review on going green in operating rooms with a reduction in cost and improvement of environmental impact [50]. Xiao et al. proposed a Roadmap for environmental sustainability of plastic use in anesthesia and perioperative areas [24]. These are some of the frameworks that demonstrate increasing alignment between federal guidance, professional societies, industry partners, research and advocacy groups, and clinical leadership.

### 2.3. Barriers to Sustainable Healthcare

Multiple barriers, such as personnel and staff, institutional buy-in, and limited regulatory enforcement, hinder the implementation of sustainable healthcare delivery initiatives. Personnel barriers are highlighted particularly by the lack of institutional leadership, staff engagement, and commitment to sustainability goals across healthcare organizations [19,51]. Without support from executive leadership and clinical champions across all organizational levels, sustainability efforts struggle to gain traction. Additionally, even within organizations where there may be personnel buy-in, staff turnover and loss of sustainability champions may hinder the progress of prior sustainability efforts [51,52].

Other barriers span institutions and systems, including competing priorities such as infection control, immediate clinical outcomes, patient safety, or cost reduction. For example, to ensure infection control or anesthesia care, some institutions may adopt single-use products, which may increase waste [53,54,55], but to optimize plastic pollution reduction, moderation is necessary [18,36,56]. While there may be momentum for reusing and recycling some equipment or products, health system regulatory requirements or patient safety concerns may further inhibit consistent adoption of sustainable practices [57].

Additionally, investing in sustainability efforts requires high upfront costs for which return on investment may not be immediately quantifiable. As such, procurement policies in many hospitals may favor lowest-cost purchasing over life-cycle value assessment, reinforcing reliance on single-use products and limiting vendor accountability for environmental impact [18]. However, multiple studies have shown that investing in sustainability may yield notable long-term savings [55,58]. For example, Sullivan et al. demonstrated cost savings in some hospitals of up to 694,140 USD per year after intervention [50].

In addition to personnel and institutional barriers, fragmented government oversight and inadequate enforcement of sustainability performance requirements may limit progress. Some of these barriers include limited standardized frameworks for measuring and reporting sustainability initiatives, as highlighted by Zurynski et al. [19], inadequate access to peer institutions’ data for monitoring and evaluation [53], and inconsistent requirements for sustainability reports as part of corporate social responsibility from large healthcare organizations [58].

## 3. Stanford Sustainability Initiatives

The Stanford Medicine supply chain system touches 31.5 million individual items annually, with over 100,000 unique medical items ordered, representing a substantial opportunity to reduce environmental emissions and waste. Stanford Medicine initiatives to improve supply chain efficiency and patient care focus on two key supply chain sustainability areas: (1) high-value care to improve the quality of care for patients while reducing cost, and (2) global impact to reduce waste and carbon production. The following are efforts that have been implemented to work towards a sustainable healthcare system. Unless otherwise cited, the outcomes reported below derive from internal Stanford Medicine program and quality-improvement data and have not been independently audited or subjected to formal life-cycle assessment.

### 3.1. Governance and Leadership

**Figure 3:**
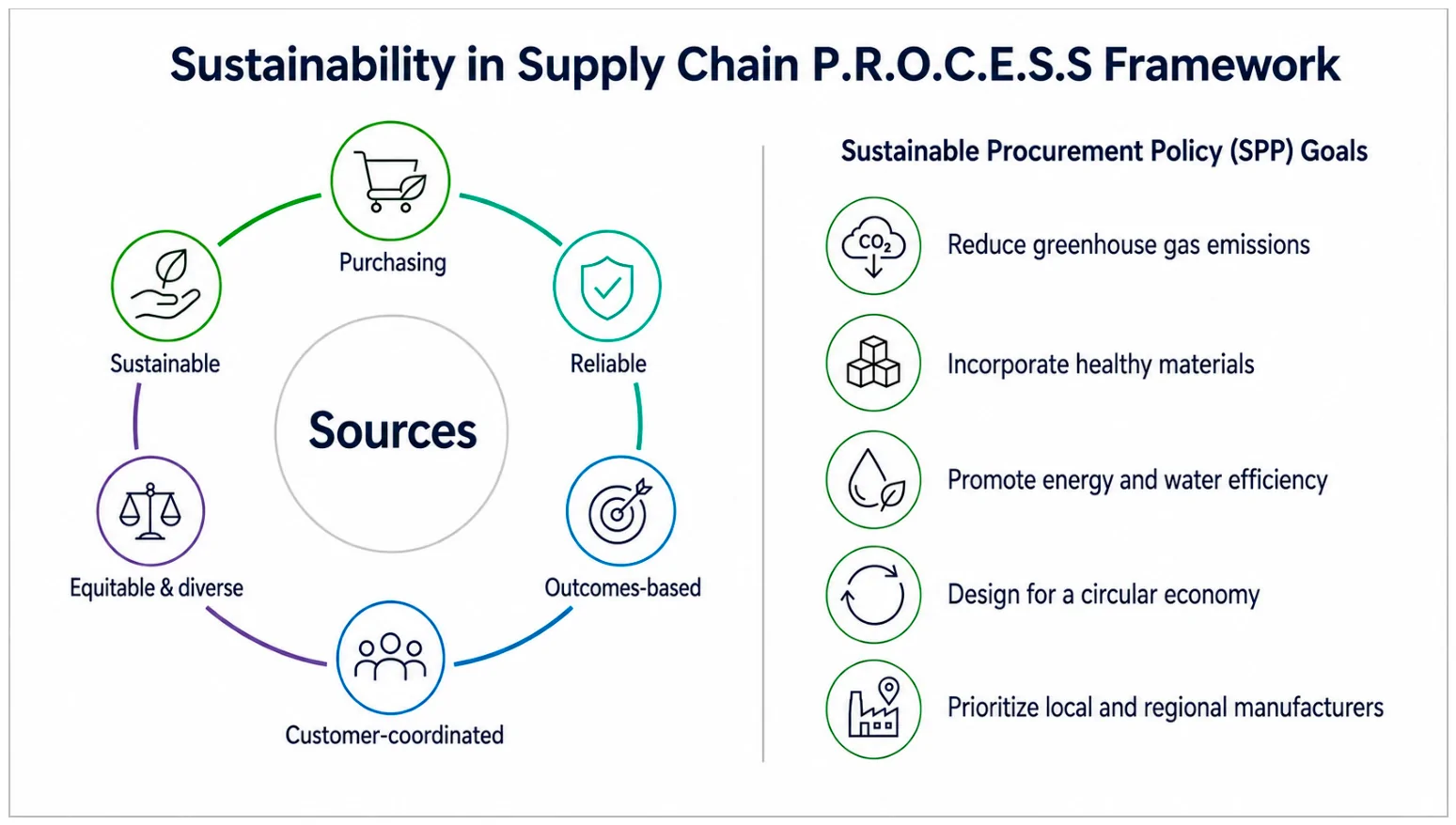
Stanford Medicine has adopted a sustainable procurement policy and supply chain embedded into the PROCESS Framework. The P.R.O.C.E.S.S. framework embeds sustainability into supply-chain decision-making by linking procurement practices with emissions reduction, healthy materials, resource efficiency, circular economy, and support for local and regional manufacturers.

Stanford Medicine has incorporated sustainability into its institutional mission through the PROCESS framework: Purchasing, Reliable, Outcomes-based, Customer-coordinated, Equitable and diverse, Sustainable Sources (Figure 3). Under the leadership of the Stanford Medicine supply chain team, Stanford Medicine established this framework to enhance the value equation of cost, quality, and service to a more comprehensive approach of decision-making, incorporating sustainability as a core measure. The value equation has been updated to incorporate the term “Outcomes-Based”. As this framework was established, Stanford developed a specific Supply Chain Sustainability Roadmap, aligning with the 2022 AHRQ Decarbonization Roadmap and California’s Climate Corporate Data Accountability Act (SB253) [59] and Climate-Related Risk Disclosure Act (SB261) [60]. The PROCESS framework and the Sustainability Roadmap operationalize sustainability across governance and procurement, embedding sustainability criteria into vendor selection, supplier accountability, and performance evaluation.

These governance changes are structural, and their effect on measured emissions or procurement outcomes has not yet been evaluated. Alignment with California disclosure statutes (SB253, SB261) also means part of this activity responds to state regulatory mandates that do not apply in most other jurisdictions.

### 3.2. Circular Procurement and Scope 3 Emission Reduction

**Figure 4:**
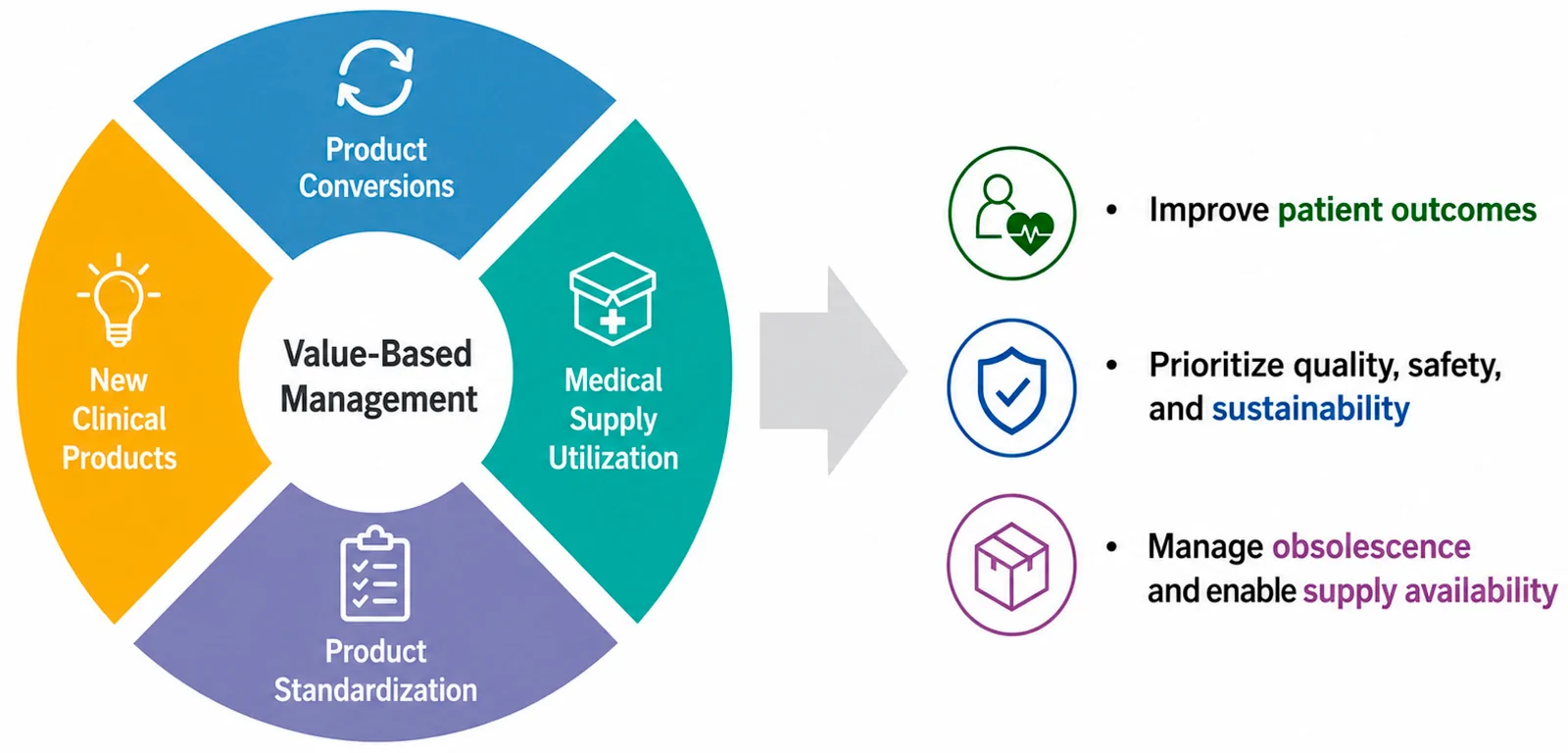
Stanford Medicine Value-Based Management (VBM) framework to assess all medical products and supplies on key sustainability attributes.

Recognizing that over 70% of emissions originate from the supply chain [9,28,61], Stanford Medicine has prioritized Scope 3 decarbonization through sustainable sourcing and product life-cycle management. Therefore, partnerships with companies and vendors whose missions and practices align with Stanford Medicine’s sustainability goals are of paramount importance. For example, Stryker Sustainability Solutions and Medline have established reprocessing and remanufacturing take-back programs, reducing single-use device waste and extending product lifespans. Stanford Medicine also instituted a Value-Based Management (VBM) program—a healthcare delivery model where providers are paid based on patient health outcomes—which requires vendors to disclose Scope 1 and 2 emissions, product recyclability, waste reduction, and chemical safety data (Figure 4). This integration of environmental metrics into purchasing decisions reflects a shift from cost-based procurement to life-cycle-based value assessment. However, vendor-disclosed emissions and recyclability data are self-reported and were not independently verified. Reprocessing and take-back programs also carry their own transport and reprocessing footprint that was not captured in a life-cycle assessment, so the reported benefit reflects diverted single-use volume rather than a net cradle-to-grave emissions accounting.

### 3.3. Clinical Integration and Waste Reduction

#### 3.3.1. Lean Surgery and Surgical Setup Reduction

A multidisciplinary surgical team led by Tevlin et al. and Garcia et al., through their Greening Hand Surgery initiative, applied the lean process improvement methods to optimize: (1) surgical field sterility, (2) surgical tray setups, and reduce operating-room waste for hand surgery and procedure packs, and (3) gown use by surgeons and OR staff [62,63]. Through thorough observation, evaluation, and team collaboration, 13 consistently unused items, including large basins, extra drapes, towels, redundant instruments, and hand surgery procedure packs, were identified and removed. This reduced not only cost ($105 saved per case) and waste (64% reduction) but also led to more rapid and efficient setups, while maintaining patient safety and workflow efficiency [62]. This approach may be adapted by other surgical departments, using department-specific data to match equipment supply to the needs of a procedure.

#### 3.3.2. Preference-Card Optimization

Orthopedic surgery is one of the specialties generating the most operating-room waste, as demonstrated by Nosrat and Swarup as well as Saleh et al. [64,65]. Sustainability champions in orthopedic surgery, such as Bellaire et al., advocated for improved awareness and reduction in operating-room waste in orthopedic surgery [66,67,68]. These efforts and commitment from Stanford Medicine leadership led to the formation of a multidisciplinary team, led by Shea et al., to improve surgical efficiency through preference-card optimization [69,70,71]. A preference card refers to a list of equipment and tools that a surgeon needs for a procedure. Surgeons often add items to preference cards over time, and they are rarely reviewed, leading to the accumulation of tools and materials set up from the list but not frequently used and therefore wasted. A hospital-wide team of surgeons, anesthesiologists, nurses, and supply chain staff reviewed surgical preference cards against actual intraoperative use and measured financial and sustainability impact. A novel reproducible algorithm was developed by Scheinker et al. to optimize preference cards, leading to an 8.38% ($352, SD $6622) reduction in mean cost of supplies per case compared to a 13.21% ($405, SD $14,706) increase for cases that did not incur preference-card optimization (*p* < 0.001) [70]. This data-driven process supports the standardization of preference cards across surgeons and procedures. The team is also integrating artificial intelligence (AI) applications to analyze procedure cost deviations and identify high-impact changes to improve efficiency and minimize waste.

The large standard deviations relative to the means (for example, a $352 mean reduction with an SD of $6622) indicate that per-case savings varied widely and were likely concentrated in a subset of cases, so the aggregate figure warrants cautious interpretation. Cost of supplies is also a proxy for environmental impact; direct waste and emissions reductions were not measured, and the AI application described remains in development without reported outcomes.

#### 3.3.3. Operating-Room Sign-Out Review

Most healthcare systems perform a surgical safety checklist to improve patient safety [72]. Stanford Medicine has added a step to this checklist during the surgical safety sign-out phase [73] (Table 1). This step involves the assessment of waste produced during the case and the identification of areas for improvement. This initiative builds on prior efforts such as the previously demonstrated “Green Moment” initiative in Ireland hospital operating rooms, where questions are asked before and after each surgical case to ensure optimal use or necessity of resources [74]. Examples include instances where fewer trays or supplies could have been used, and whether preference card changes are needed to prevent similar waste in future cases. This structured reflection uses routine safety debriefs as a prompt for sustainability review, ensuring that everyone in the team continues to participate in upholding a more sustainable operating or procedure room environment [74,75,76]. It engages all healthcare providers to participate and become active thinkers and stakeholders in devising solutions to promote sustainability.

#### 3.3.4. Partnership with Major Supply Chain Industry

Stanford Medicine has collaborated with a major supply chain industry partner to streamline surgical kits by removing redundant tools and using the prior optimization of preference cards to incorporate single-use device remanufacturing where clinically appropriate [77]. Some of the items that were identified and removed from the preference cards include suction canister liners, emesis basins, and plastic bins [78]. This partnership extends device life cycles, decreases procurement frequency, and diverts equipment and tools that were going to be, or contribute to, waste away from landfills. This collaboration provides another opportunity to reduce the Stanford Medicine supply chain footprint. Scaled across many operating rooms and departments, such collaboration may provide significant environmental and financial benefits [79,80].

#### 3.3.5. Recycling Operating-Room Plastics

This OR staff-led initiative targets one of the largest and most valuable waste streams in the operating room: high-value recyclable plastics. The peer-reviewed literature and waste audits confirm that 60–74% of non-infectious OR waste—primarily packaging, plastics, and disposables—can be captured for recycling if robust separation programs are implemented [81,82,83]. The key lies in identifying and segregating high-value plastics at the source: #1 polyethylene terephthalate (PETE), #2 high-density polyethylene (HDPE), and #5 polypropylene (PP), including blue wrap [82,84].

The Hospital Plastics Characterization and Recycling Feasibility Study found that with targeted separation practices, 60–70% of plastics in hospital waste—including surgical trays, IV bags, packaging, and containers—could be recycled, with the OR identified as a key opportunity due to the high concentration of clean, single-use items [81]. European and U.S. pilot studies found that #1 (PETE) and #2 (HDPE) plastics—alongside polypropylene and packaging—compose more than 50% of the recyclable content in surgical waste audits, especially when plastics are segregated at source by staff [82,83,85]. Blue wrap alone accounts for 11–25% of total OR waste by weight and can be almost entirely diverted from landfill through dedicated collection systems [23,82,86].

A corollary of the “plastic straw moment,” polypropylene plastics are easily recognizable by most staff. By collecting these notable high-value plastic items every day, every team and staff member can participate in reducing waste. This low-cost intervention has engaged staff in source separation of high-value plastics and expanded participation in sustainable operating practices in the OR [84].

#### 3.3.6. Green Anesthesia and Nitrous Oxide Leak Reduction

The Green Anesthesia initiative focuses on reducing greenhouse gas emissions from volatile anesthetics. Nitrous oxide is one of the most harmful anesthesia gases, with very high global warming potential, over 250 times more potent than carbon dioxide as a greenhouse gas, and unlike other anesthetics, lingers in the atmosphere for over a century, destroying the ozone layer [47]. However, the biggest nitrous oxide challenge is not the gas administered to patients. It is the systemic leakage from the hospital pipes. Up to 99% of nitrous gas supply is lost through hidden leaks from pipeline valves or at central storage sources [87,88], before reaching the operating room, contributing to more than 80–90% of global anesthetic gas emissions [88,89,90].

Because of this challenge, the American Society of Anesthesiologists recommends replacing central piped nitrous oxide with portable e-cylinders [88,91]. This intervention was implemented by Kraybill et al. at Stanford [92]. With sustainability champions, the full transition from central pipeline to portable e-cylinders was completed in 6 months, reducing nitrous oxide leakage and associated costs through improved gas efficiency.

Besides the infrastructure redesign, Stanford Medicine also instituted ongoing educational training for anesthesiologists and staff, consistent with the published literature, in partnership with CODA, a medical education and health promotion charity that aims to deliver climate action-oriented recommendations, supported by useful resources and success stories [93]. The education and training included choosing anesthetic gases that are less harmful to the environment, being conscious about delivering sustainable anesthesia care, including using less nitrous oxide overall, adopting low-flow carrier gas machine settings, and leveraging electronic health record data to monitor the anesthesiology department’s greenhouse gas emissions. These efforts contribute to the broader strategy to reduce Stanford Medicine’s Scope 1 emissions and create a model of sustainable anesthesia practice.

#### 3.3.7. Addressing World Inequity via Sustainable Healthcare (AWISH) Program

The AWISH program, founded and directed by Caroline Lin, MD, a sustainability champion, is a fusion of staff and community volunteer sustainability program at Stanford Medicine [94]. AWISH was created to confront two interconnected challenges: the environmental burden of medical waste and the global inequities in access to medical supplies. Hospitals in high-income countries discard unused but unopened supplies or tools that could be used to deliver care in resource-limited settings. AWISH addresses this problem through established workflows to recover, quality-check, and redistribute these supplies, turning what would have been medical waste into a valuable resource to deliver care where needed. A prior study based on the Supporting Hospitals Abroad with Resources and Equipment (SHARE) program at Johns Hopkins University demonstrated that approximately 2 million lbs per year (907,185 kg) of medical supplies were recovered from operating rooms, with a donation value of up to US $15 million per year, and a cost-utility of US $2.14 per DALY averted [95]. The AWISH program builds on the legacy of the SHARE program [95] and other pioneering programs such as Recovered Medical Equipment for the Developing World at Yale University (REMEDY) [96,97], which demonstrated that volunteer-driven recovery of unused medical materials can help reduce waste and meaningfully improve health outcomes in underserved communities.

AWISH began as a seed-funded pilot within Stanford’s sustainability program and has currently grown into a hospital-wide effort that engages frontline clinicians, supply-chain staff, and a large volunteer network of students, consistent with prior models of volunteer-based programs [98,99]. AWISH now has defined workflows, audit tools, and education programs that engage frontline teams and connect departments. Some key initiatives include the recycling of the blue wrap used to cover surgical instrument trays to keep them sterile prior to use. The initiative diverts clean, uncontaminated sterile wrap, which accounts for more than 20% of OR waste and transforms it through community partners into reusable products. More than 5000 pounds to date, and nearly 3 tons annually, of blue wrap have been diverted since 2024.

AWISH has also championed other sustainability initiatives. For example, the collection of used LigaSure devices generates approximately $22,000 annually in rebates. Through the dedication of student volunteers, who contributed over 2000 h in 2025, AWISH saved the hospital nearly $74,000 in labor costs. Another volunteer-run pediatric intensive care unit (PICU) reshelving project reported the following outcomes: cart costs were reduced by 71%, cart weight by 53%, and combined savings from recovered supplies and optimized inventory exceeded $1.2 million per year.

The AWISH program has expanded into labor and delivery services and other high-utilization units, contributing to hospital-wide circular supply chain efforts. Through these interconnected initiatives, AWISH demonstrates how hospital leadership, staff and community engagement, system redesign, and equitable resource distribution can advance sustainability, reduce waste, and promote global health equity. Future projects include scaling plasma sterilization and expanding specialized donations to more partner hospitals.

## 4. Discussion

In this article, we examine strategies that have produced benefits in healthcare sustainability, barriers that have hindered adoption and progress, and the impact of governance, supply chain management, and personnel engagement may be integrated within a complex health system. The literature highlights key areas to reduce emissions, including Scope 3 emissions, energy-intensive infrastructure, anesthetic gases, single-use materials, and operating-room waste [9,12,13,14,15,16]. However, effective implementation of sustainability measures remains challenging due to limited regulatory accountability, fragmented healthcare and hospital systems, low institutional leadership buy-in and staff training, lack of vendor transparency, and competing clinical interests [51,52,53,54,55,56,57]. Therefore, to enact significant changes, sustainability challenges in healthcare should be addressed at an organizational level rather than focusing on a few key areas, such as recycling or a single waste reduction project.

The model of Stanford Medicine illustrates how sustainability efforts may be incorporated at an institutional level, encompassing leadership buy-in, supply chain, surgical and anesthesiology infrastructure, and waste management systems consistent with prior systematic, scoping, and narrative reviews of sustainability initiatives for operating rooms and hospital systems more broadly [9,75,76,86]. The initiatives at Stanford serve to demonstrate how leadership coordination, supply chain redesign, clinician champions, industry and vendor partnerships, as well as individual staff participation, may lead to lasting institutional change. Nevertheless, these initiatives are described in a complex, well-resourced setting, and they vary in maturity.

Given differences in resources across institutions, the described initiatives should be viewed as institutional implementation examples that may inspire change in other institutions rather than blueprints for universal sustainable healthcare scalability. For example, reusable or reprocessed products may reduce waste. However, these efforts must be balanced with validated infection-prevention protocols, cleaning capacity, regulatory and institutional oversight, and life-cycle assessment. Recycling and donation of unused products must be conducted in a dignified fashion, ensuring that donated materials meet the standards of donor and recipient countries. Low-cost initiatives that may be adaptable include staff education, preference-card optimization, and sign-out prompts. Conversely, other measures, such as establishing departments for emission reductions, hospital renovations, and supply chain vendor negotiations, may require resources that small or low-resource settings may not possess.

While systems in high-resource settings like Stanford Medicine offer many lessons, they can also learn from other systems. For example, only 2.2% of surveyed U.S. perioperative professionals reported reusing anesthesia breathing circuits, compared with 71–76% in other high-, upper-middle-, and lower-middle-income countries [100]. Reusable circuits used with appropriate bacterial and viral filters have not demonstrated increased infection risk and may reduce cost, water consumption, energy use, and waste [56,101,102,103]. The Pall/Cytiva Ultipor 25 system is FDA-cleared for 24 h multipatient use with replacement of the patient-specific filter [56]. Because anesthesia may account for up to 25% of operating-room waste and circuits may constitute 60–80% of anesthesia waste volume, circuit reuse represents a potentially high-impact intervention [7,104]. Similar adoption gaps exist in device reprocessing and institutional certification: approximately 120 U.S. hospitals had achieved The Joint Commission’s Sustainable Healthcare Certification by 2025 [105], whereas Norwegian hospitals operate under certified environmental management standards [106], and ISO 14001 has been adopted widely internationally [107,108]. These comparisons require caution because regulatory and financing systems differ, but they highlight the need for future studies and interventions to generate nationally representative data, inform international benchmarking, and inform research on the regulatory, financial, cultural, and manufacturer-related barriers affecting adoption. Future studies may also ensure the collection of data using standardized sustainability performance metrics such as carbon footprint per procedure, waste per bed, and cost per intervention, in addition to previously published key performance indicators by Dolcini et al. [109].

Based on the reviewed literature, lessons from the Stanford Medicine experience, and the Exploration, Preparation, Implementation, Sustainment (EPIS) framework, an implementation-science model that organizes adoption across four phases while accounting for organizational context, external policy and market conditions, intervention characteristics, and cross-sector partnerships, we propose the recommendations summarized below (Table 2) [110].

## 5. Limitations

This article has several limitations. Despite a detailed literature review and synthesis, as a perspective, this article does not provide the exhaustive search or reproducibility of a systematic review, and some studies may have been omitted. The included literature is heterogeneous in design, setting, outcome definitions, denominators, and carbon-accounting methods, limiting direct comparison and data analysis. The Stanford Medicine model represents a well-resourced quaternary academic health system with substantial institutional, procurement, technical, and volunteer capacity. As such, its findings may not be directly transferable to limited-resource settings. Some reported outcomes derive from observational or quality-improvement initiatives without concurrent controls, full life-cycle assessment, standardized emissions measures, or long-term follow-up. Additionally, several authors participated in the initiatives described, introducing potential selection and positive-reporting bias. Accordingly, the Stanford initiatives should be viewed as implementation examples rather than evidence of causality, comparative effectiveness, or universal scalability.

## 6. Conclusions

The healthcare system contributes significantly to environmental pollution, and concerted efforts to integrate sustainability into health system design and healthcare delivery are critically important. International organizations, governments, and advocacy groups have provided frameworks and guidelines to reduce emissions and waste across health systems. However, personnel, financial, and institutional cultural barriers may hinder widespread adoption of sustainable healthcare delivery. Stanford Medicine initiatives illustrate that reducing waste, reprocessing devices, and eliminating high-emission anesthetic gases provide improvements in reducing the cost of care and pollution on the environment. Academic medical centers and other healthcare institutions can benefit significantly from collaboration with industry, government, and community stakeholders, as these key partners provide essential resources, expertise, and support systems to ensure the success and sustainability of initiatives.

## Figures and Tables

**Table 1:** Stanford Medicine operating-room checklist. The checklist demonstrates the sign-out questions to improve sustainable use of operating-room resources.

Operating-Room Sign-In	Time-Out	Sign-Out
Led by: Nurse/Technologist/Anesthesiologist before induction	Led by: Circulator after preparing and draping the surgical site	Led by: Circulator
□ Patient band check	□ Patient (Name, Medical Record Number, Date of birth)	□ Procedure performed
□ Allergies	□ Verify procedure/consent(s)	□ Wound class
□ Weight	□ Correct site/site mark visible	□ Specimens identified and labeled
□ Consented procedure	□ Allergies	□ Completion of counts
	□ Antibiotics	□ Questions/concerns
	□ Imaging, implants, and equipment	• Equipment issues?
	□ Fire risk	• Fewer trays/supplies?
	□ Concerns: duration, disposition, blood loss	• Preference card changes?
	□ Introductions	

**Table 2:** EPIS-guided pathway for sustainable healthcare delivery. Patient safety, infection prevention, clinical effectiveness, and health equity should remain balancing measures throughout all EPIS phases.

EPIS Phase	Recommended Actions	Core Measures
**Exploration**	Identify priority emissions and waste sources, assess organizational readiness, clinical risks, and intervention fit.	Scope 1–3 emissions, waste per case or patient-day, readiness and feasibility
**Preparation**	Establish governance, engage clinicians, procurement, infection prevention, facilities, and frontline staff, define targets and workflows.	Accountable leads, implementation plan, baseline targets, stakeholder participation
**Implementation**	Optimize preference cards and supply kits, reduce high-impact anesthetic gases, and introduce safe reuse, reprocessing, recycling, and recovery programs.	Adoption and fidelity, avoided waste and emissions, cost and patient-safety outcomes
**Sustainment**	Integrate successful practices into policies, contracts, budgets, training, and routine performance reporting.	Long-term target attainment, maintained savings, staff participation, and intervention durability
**Context and bridging factors**	Evaluate regulation, accreditation, vendor relationships, financing, institutional capacity, and partnerships throughout all phases.	Barriers, facilitators, adaptations, equity, and scalability across hospital types

## Data Availability

The original contributions presented in this study are included in the article. Further inquiries can be directed to the corresponding author.

## Abbreviations

The following abbreviations are used in this manuscript:

WHO	World Health Organization
AHRQ	Agency for Healthcare Research and Quality
SAGES	Society of American Gastrointestinal and Endoscopic Surgeons
EAES	European Association for Endoscopic Surgery
WFSA	World Federation of Societies of Anesthesiologists
EPA	United States Environmental Protection Agency
ASA	American Society of Anesthesiologists
AWISH	Addressing World Inequity via Sustainable Healthcare
SHARE	Supporting Hospitals Abroad with Resources and Equipment
REMEDY	Recovered Medical Equipment for the Developing World
OR	Operating Room
PICU	Pediatric Intensive Care Unit
IV	Intravenous
FDA	Food and Drug Administration
AI	Artificial Intelligence
DALY	Disability-Adjusted Life Year
SD	Standard Deviation
GHG	Greenhouse Gas
CO_2_	Carbon Dioxide
Mt	Million metric tons
PETE	Polyethylene Terephthalate
HDPE	High-Density Polyethylene
PP	Polypropylene
PROCESS	Purchasing, Reliable, Outcomes-based, Customer-coordinated, Equitable and diverse, Sustainable Sources
VBM	Value-Based Management
SB253	California Climate Corporate Data Accountability Act
SB261	California Climate-Related Risk Disclosure Act
USD	United States Dollar
EPIS	Exploration, Preparation, Implementation, Sustainment
ISO	International Organization for Standardization
